# Identification of Immune-Related Genes and Small-Molecule Drugs in Interstitial Cystitis/Bladder Pain Syndrome Based on the Integrative Machine Learning Algorithms and Molecular Docking

**DOI:** 10.1155/2022/2069756

**Published:** 2022-12-28

**Authors:** Yiheng Jiang, Xinqing Zhu, Abdullah Y. Al-danakh, Qiwei Chen, Deyong Yang

**Affiliations:** ^1^Department of Urology, First Affiliated Hospital of Dalian Medical University, Dalian 116021, China; ^2^School of Information Science and Technology of Dalian Maritime University, 116000, Dalian City, Liaoning Province, China

## Abstract

**Background:**

Interstitial cystitis/bladder pain syndrome (IC/BPS) is a chronic, severely distressing clinical syndrome characterized by bladder pain and pressure perceptions. The origin and pathophysiology of IC/BPS are currently unclear, making it difficult to diagnose and formulate successful treatments. Our study is aimed at investigating the role of immune-related genes in the diagnosis, progression, and therapy of IC/BPS.

**Method:**

The gene expression datasets GSE11783, GSE11839, GSE28242, and GSE57560 were retrieved from the GEO database for further analysis. Immune-related IC/BPS differentially expressed genes (DEGs) were identified by limma. Three distinct machine learning approaches, least absolute shrinkage and selection operator (LASSO), support vector machine–recursive feature elimination (SVM-RFE), and random forest (RF), were used to find the immune-related IC characteristic genes. Nomogram and receiving operator curves (ROC) were plotted to measure characteristic effectiveness. Using the CMap database and the molecular docking approach, potential small-molecule medicines were found and verified. Consensus cluster analysis was also performed to separate the IC/BPS samples into immunological subtypes.

**Results:**

A total of 24 immune-related IC/BPS-DEGs were identified. When compared to the normal control group, the IC/BPS cohort had significantly more immune cell infiltration. Integrative machine learning methods discovered 5 IC/BPS characteristic genes (RASGRP1, PPBP, RBP4, CR2, and PROS2) that may predict IC/BPS diagnosis and immune cell infiltration. Furthermore, two immunological subgroups with substantial variations in immune cell infiltration across IC/BPS samples were identified, which were named cluster1 and cluster2, with the hallmark genes having greater expression in cluster2. Finally, bumetanide was shown to have the potential to be a medication for the treatment of IC/BPS, and it performed well in terms of its molecular binding with RASGRP1.

**Conclusion:**

We found and validated 5 immune-related IC/BPS genes (RASGRP1, PPBP, RBP4, CR2, and PROS2) and 2 IC/BPS immune subtypes. In addition, bumetanide was discovered to be a potential drug for treating IC/BPS, which may provide new insight into the diagnosis and immune therapy of IC/BPS patients.

## 1. Introduction

Interstitial cystitis/bladder pain syndrome (IC/BPS) is a chronic inflammatory disorder that has a high degree of heterogeneity. It is frequently accompanied by severe pelvic pain and symptoms of the lower urinary tract, and it has a negative impact on the quality of life for millions of people [1]. The European Society for the Study of Interstitial Cystitis (ESSIC) defines IC as an unpleasant urinary bladder feeling that lasts for more than six months and is accompanied by at least one lower urinary tract symptom that lasts for more than six weeks. It is estimated that 7.9 million women and 2 million men in the United States alone are affected by IC, which results in an annual cost of more than 750 million US dollars [2, 3]. However, despite the significant social and economic implications of IC/BPS, the unavailability of IC/BPS etiology and pathophysiology complicates the process of its diagnosis and treatment [4]. Because oral medicines for IC/BPS are often ineffectual, the major goal of current treatment is to reduce the severity of the condition's symptoms [5]. Approximately 10% of IC/BPS complicated cases need invasive surgical therapy, such as improved bladder capacity through ileal cystoplasty and urinary bladder diversion surgeries [6, 7], which certainly increases the physical and emotional impact on patients. Therefore, the development of novel molecular biomarkers for IC/BPS diagnosis and therapy is urgently required.

Evidence from epidemiology showed that chronic inflammatory responses and the immune system both play an important part in the development of IC/BPS [8]. Previous studies have demonstrated aberrant activity of numerous immune cells in the IC tissue samples [9] as well as higher levels of proinflammatory in urine and serum samples of IC patients [10]. Although there is yet no adequate evidence to indicate that IC is an autoimmune illness, the aberrant immunological state of IC has been clearly documented in various research [11–13]. Previous bioinformatics studies using single-cell RNA sequencing found that local adaptive immune responses are a hallmark of IC [6], and Gamper et al. [14] reported that immune-related pathways and immune cell infiltration were involved in the initiation and progression of IC/BPS, highlighting the pivotal role of immune mechanisms in the disease. Therefore, in this study, we integrated three different machine learning algorithms, including LASSO, SVM-RFE, and RF, to identify immune-related IC/BPS characteristic genes that might aid in the assessment of immune state in IC/BPS patients and facilitate their diagnosis. Moreover, we proposed novel immune subtypes of IC/BPS samples containing high and low immune infiltration. Finally, the CMap database was used to identify small-molecule medicines for treating IC/BPS based on immune-related IC/BPS signature genes; further molecular docking also corroborated the proposed mechanism of action.

## 2. Materials and Methods

### 2.1. Study Cohorts

The GEO database was searched for IC/BPS and used to download four independent public datasets (GSE11783, GSE11839, GSE28242, and GSE57560); the detailed dataset information is shown in Supplementary Table 1. Because of the differences in sequencing platforms, methods, and experimental designs that exist between these previously mentioned datasets, the combat function was utilized to eliminate batch effects that existed between samples. This function was based on the SVA package that is included in the R programming language. In the end, a meta-GEO cohort consisting of 37 IC samples and 20 normal control samples was produced for the sake of further investigation. Principal component analysis (PCA) was used to visualize the performance of debatching. Integrative gene expression profiles from all GEO datasets were used to establish a coexpression network and identify immune-related IC/BPS characteristic genes.

### 2.2. Analysis of Immune-Related IC/BPS-DEGs

Firstly, to identify IC/BPS differentially expressed genes (DEGs), a differential analysis was performed between 37 IC/BPS samples and 20 normal control samples using the R package limma (the cutoff score was set as*p* < 0.05 and absolute (log 2 FC) > 1.0) and was conducted to select IC/BPS differentially expressed genes (DEGs). Following that, we obtained the immune gene list from the ImmPort database [15]. Subsequently, we found the genes intersecting between immune-related genes and IC/BPS-DEGs; these matched genes were designated immune-related IC/BPS-DEGs.

### 2.3. Gene Functional Enrichment Analysis and Gene Set Variation Analysis (GSVA)

The functional enrichment analysis of the preceding gene list was performed primarily using the R package ClusterProfiler. Genes were uploaded to the Gene Ontology (GO) and the Kyoto Encyclopedia of Genes and Genomes (KEGG) databases to elucidate essential molecular processes and biological pathways further [16, 17]. In addition, we assessed the biological importance of the IC characteristic genes using the R package GSVA based on the fifty hallmark gene sets from the molecular characteristic database MSigDB [18].

### 2.4. Construction of Protein-Protein Interaction (PPI) Network and Identification of Hub Genes

In order to investigate how the expression of protein-coding genes is interacted with each other, immune-related IC/BPS-DEGs were uploaded to the STRING database [19]. The PPI network was then constructed with the cutoff score set at 0.400. Subsequently, the information on gene interactions was analyzed based on the Molecular Complex Detection (MCODE) tools in Cytoscape; hub modules (genes) in the PPI network were found by applying the criteria of degree cutoff = 2, node score cutoff = 0.2, and *K* − Core = 2.

### 2.5. Identification and Validation of Potential Signature Genes

In order to select immune-related IC/BPS characteristic genes, the LASSO, RF, and SVM-RFE machine learning techniques were used. It generates a more refined model by generating a penalty function, which compresses certain regression coefficients and requires the total of absolute values of coefficients to be smaller than a set value. The LASSO regression model is a compressed estimate model. LASSO analysis was implemented based on the R package glmnet [20]. A random forest is a classifier consisting of numerous decision trees, and the mode of the category output determines its output category by the individual tree [19]. The mean decrease accuracy of each gene was ranked using recursive feature elimination in the RF model; the top 10 genes were recognized as signature genes. In this study, the RF model was developed using the R package random forest. SVM-RFE is a novel method for pattern recognition that adopts the principle of structural risk minimization (SRM), accounts for training error and generalizability, and demonstrates distinctive advantages in solving small samples, high-dimensional nonlinearity, local minima, and other pattern recognition problems. In this research, the SVM-RFE method was implemented using the R package kernlab [21]. In addition, the ROC curve was generated to assess the precision of the prediction findings.

Nomogram was plotted via R package rms to evaluate the characteristic value of immune-related IC/BPS characteristic genes. Furthermore, we used a calibration curve to estimate the accuracy and robustness of the nomogram prediction.

### 2.6. Consensus Clustering

On the basis of the gene expression profile of the immune-related IC/BPS-DEGs, consensus clustering, a resampling-based technique, was used to identify further clusters. The procedure was carried out using the ConsensusClusterPlus R program. The best number of clusters was estimated using CDF curve, consensus score matrix, Nbclust, and PAC score in a synthetic manner.

### 2.7. Potential Small-Molecule Drug Identification and Molecular Docking Verification

Connectivity Map (CMap) is an expression profile database that utilizes cellular responses to perturbations to identify possible functional linkages between diseases, genes, and therapeutics [22]. We uploaded immune-related IC signature genes to the CMap database in order to identify possible small-molecule medicines for the treatment of IC/BPS. From the PubChem database, the molecular findings of the active components were acquired. Subsequently, AutoDock Vina was used to conduct molecular docking of possible small-molecule medicine active components and IC/BPS main target proteins; the accuracy of IC/BPS medications was determined by the amount of binding free energy and displayed using PyMOL.

### 2.8. Statistical Analysis

All statistical analyses, data processing, and figure plotting are carried out in R 4.1.1 software. Correlation analysis was carried out using the R program ggplot2 and the Pearson correlation coefficient. To compare continuous variables, the Wilcoxon rank sum test or *T*-test was utilized. The R package pROC was used to predict binary classification variables. *p* < 0.05 was considered statistically significant.

## 3. Results

### 3.1. Identification of Immune-Related Differentially Expressed Genes in IC/BPS

**Figure 1:**
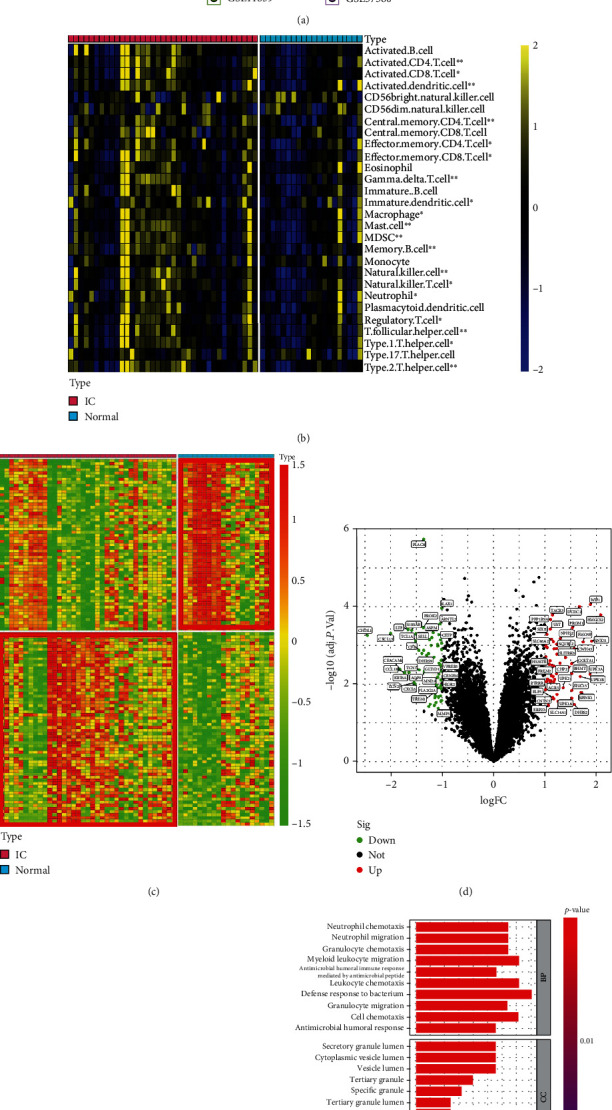
Identification of immune-related IIC-DEGs based on the combined GEO datasets. (a) The upper PCA plot performs the combined gene expression datasets of GSE11783, GSE11839, GSE28242, and GSE57560. The lower PCA plot performs the combined gene expression datasets of GSE11783, GSE11839, GSE28242, and GSE57560 after removing batch effects. (b) Abundance of 28 infiltrated immune cells evaluated by ssGSEA for IC cohort and normal control cohort. (c) The heatmap performs the differentially expressed genes between the IC cohort and normal control cohort. (d) The volcano plot shows the detailed information of the IC-DEGs. (e) The Venn diagram performs the intersection genes between IC-DEGs and immune-related genes. (f) GO enrichment analysis based on the immune-related IC-DEGs. (g) KEGG enrichment analysis based on the immune-related IC-DEGs.

In accordance with our methodological approach, we initially obtained and integrated four distinct public datasets (GSE11783, GSE11839, GSE28242, and GSE57560). After data preprocessing and batch effect removal across samples (Figure 1(a)), we merged a meta-GEO cohort encompassing 15,401 gene expression profiles from 20 normal control and 37 IC/BPS samples. To further investigate the functions of immune-related genes in IC/BPS patients, the infiltration of 28 immune cells was analyzed using single-sample gene set enrichment analysis (ssGSEA). Immune cell infiltration was significantly different between the IC/BPS and the control groups, with the IC/BPS group exhibiting a much greater immune cell infiltration abundance than the normal one (Figure 1(b)). The differential analysis of the gene expression differences revealed 117 DEGs, comprising 55 upregulated and 62 downregulated genes (Figures 1(c) and 1(d)). The intersection of 117 IC/BPS-DEGs and 1,793 immune-related genes retrieved from the ImmPort database was then used to identify a total of 24 immune-related IC/BPS-DEGs (Supplementary Table 2). In addition, functional enrichment analysis demonstrated that these immune-related IC-DEGs were intimately associated with immune biological processes and pathways, including cytokine-cytokine receptor interaction, IL-7 signaling, and chemokine signaling pathways (Figures 1(f) and 1(g)).

### 3.2. Hub Immune-Related IC/BPS-DEG Identification via PPI Network

**Figure 2:**
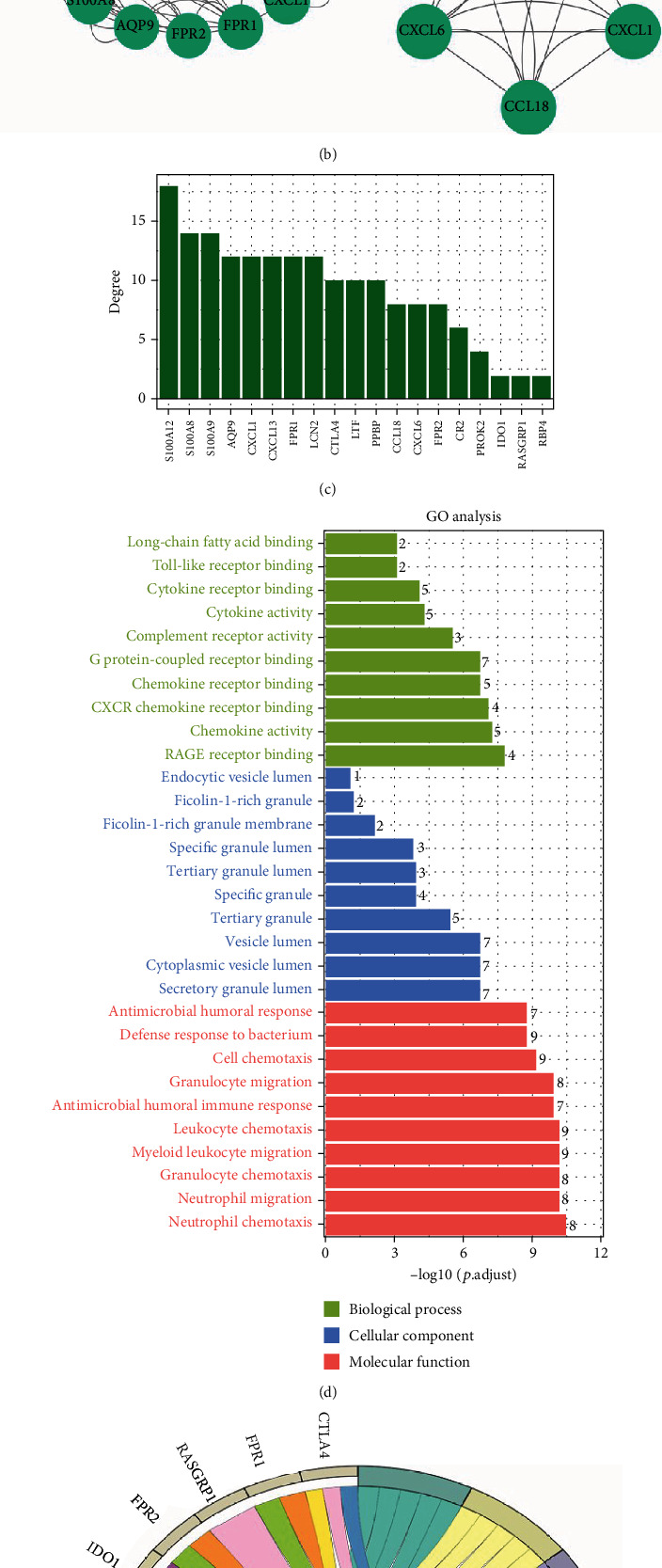
Construction of protein-protein interaction network and identification of hub immune-related IC-DEGs. (a) Protein-protein interaction network of 24 immune-related IC-DEGs. (b) Left: identification and visualization of 19 hub based on the Molecular Complex Detection (MCODE) tools in Cytoscape. Right: MCODE analysis showed the most active modules. (c) The bar plot shows the degree value of the 19 hub genes. (d) GO enrichment analysis based of the 19 hub genes. (e) KEGG enrichment analysis based of the 19 hub genes.

First, we investigated the PPI network of immune-related IC-DEGs using the STRING database (Figure 2(a)), and then, we imported the generated PPI network into the Cytoscape software, which reveals the interaction relationship of hub immune-related IC-DEGs, with nodes of hub genes arranged by degree value (Figures 2(b) and 2(c)). Furthermore, the MCODE analysis revealed which modules were the most active. The majority of these hub genes were shown to be involved in the IL-17 signaling pathway, cytokine-cytokine receptor interaction, T cell receptor signaling pathway, cytokine activity, and chemokine receptor binding. This resulting data implies that these hub genes are critical in the immunological response.

### 3.3. IC/BPS Characteristic Genes Selected via Integrative Machine Learning Algorithms

**Figure 3:**
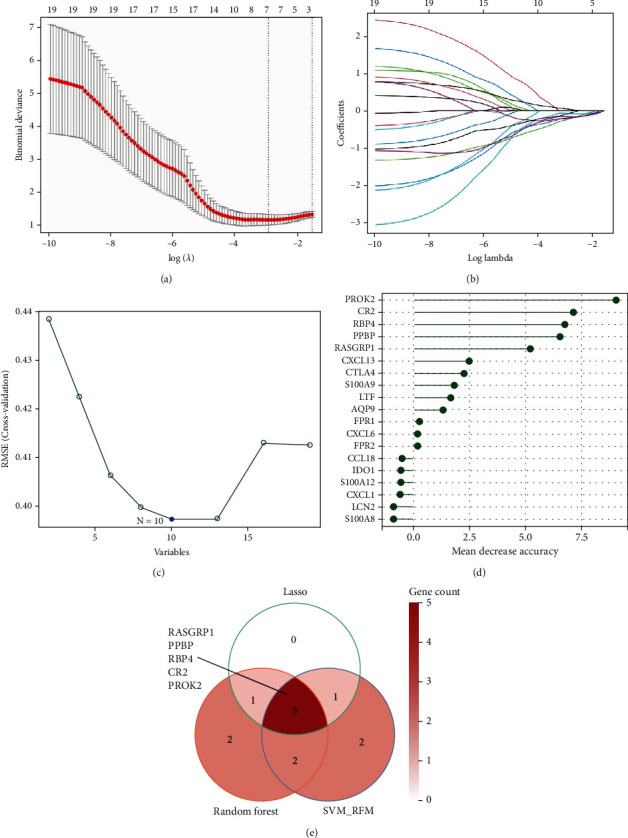
IC characteristic genes selected based on the integrative machine learning algorithms. (a, b) Identification of the IC characteristic genes using LASSO regression algorithm; the optimal lambda was determined as 0.053 after tenfold cross-validation. (c) Identification of the IC characteristic genes using SVM-RFE algorithm; the classifier showed the minimum error when *N* = 10. (d) Identification of the IC characteristic genes using random forest algorithm; the bar plot shows the mean decrease accuracy of each gene; the top 10 most important genes were selected as characteristic genes. (e) The Venn diagram performs the five most important IC characteristic genes.

Three machine learning algorithms (including LASSO, SVM-RFE, and random forest) were then integrated to select IC/BPS characteristic genes for subsequent characteristic value evaluation and nomogram construction. Following tenfold cross-validation that identified seven signature genes, the optimal value of lambda for the LASSO regression technique was found to be 0.53. (Figures 3(a) and 3(b)). For the SVM-RFE algorithm, the classifier showed the minimum error when *N* = 10 (Figure 3(c)). We also established a random forest model and determined the mean decrease accuracy for each gene; as a result, we chose the 10 most significant genes as signature genes (Figure 3(d)). Finally, the Venn diagram showed the five most important IC/BPS characteristic genes (RASGRP1, PPBP, RBP4, CR2, and PROS2) shared by these three machine learning algorithms (Figure 3(e)).

**Figure 4:**
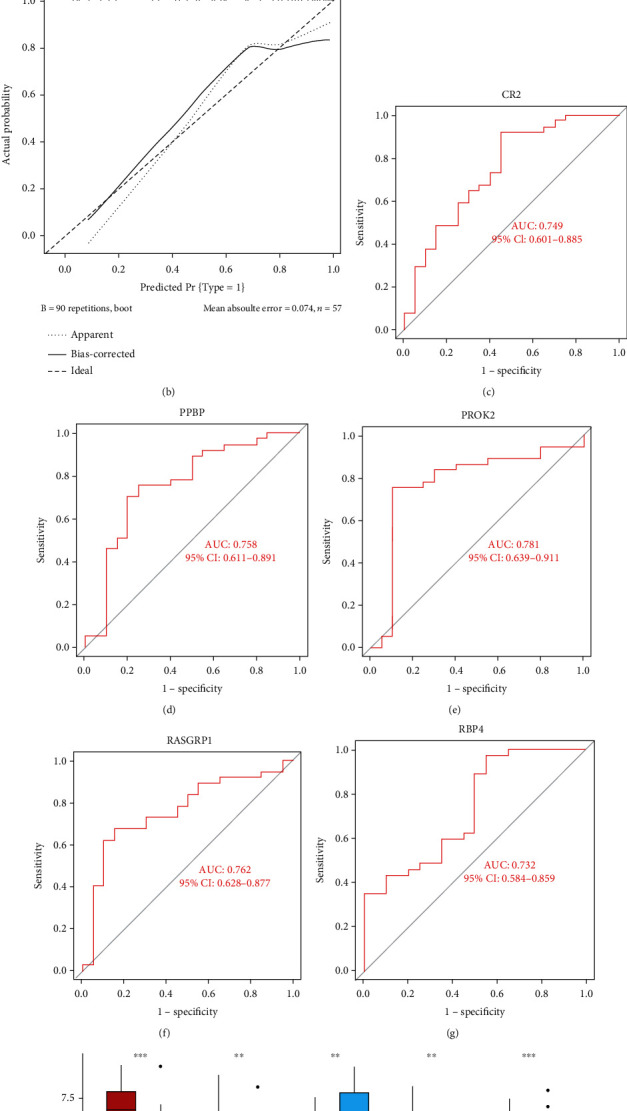
Establishment of nomogram and assessment of characteristic value. (a) Nomogram was constructed for diagnosing IC. (b) Calibration curve was plotted to evaluate the stability of the prediction results of the nomogram. (c–g) ROC curve was established to estimate the reliability and robustness of IC characteristic genes in the diagnosis of IC. (h) The box plot performs the expression of IC characteristic genes.

The nomogram was developed in order to offer clinicians a quantitative tool for risk prediction in IC/BPS patients. Every IC/BPS characteristic gene expression corresponds to a point in the nomogram. The sum of IC/BPS characteristic gene points was used to get the overall number of points representing the risk prediction percentage Figure 4(a). In addition, the calibration curve was produced to test the stability of the nomogram's prediction findings (Figure 4(b)). Moreover, ROC curves were built to determine the reliability and robustness of IC/BPS characteristic genes in diagnosing IC/BPS, with the AUC score and 95% confidence interval (CI) obtained for each gene. All the IC/BPS characteristic genes performed high characteristic value in predicting IC/BPS, CR2 (AUC: 0.749, 95% CI: 0.601-0.885), PPBP (AUC: 0.758, 95% CI: 0.611-0.891), PROK2 (AUC: 0.781, 95% CI: 0.639-0.911), RASGRP1 (AUC: 0.762, 95% CI: 0.628-0.877), and RBP4 (AUC: 0.732, 95% CI: 0.584-0.859) (Figures 4(c)–4(g)). Except for RBP4, which is significantly expressed in the control group, all genes are strongly expressed in the ICI group (Figure 4(h)).

### 3.4. Immune Status of IC/BPS Patients

**Figure 5:**
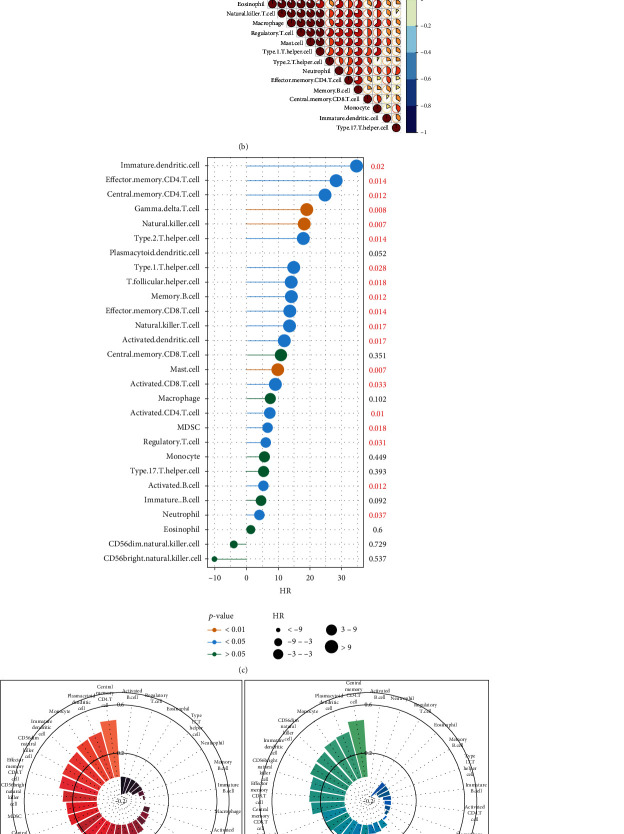
Immune status of IC. (a) The violin plot shows that both adaptive and innate infiltrated immune cells performed abundant enrichment in the IC cohort compared with the normal control cohort. (b) Correlation analysis revealed the remarkable interactions between 28 infiltrated immune cells. (c) The logistic regression model also showed that most of the immune cells were positively correlated with the diagnosis of IC. (d) The radar plot visualized the scores of infiltrated immune cells. (e) Correlation analysis of infiltrated immune cells and IC characteristic genes. ^∗^ represents *p* < 0.05, ^∗∗^ represents *p* < 0.01, and ^∗∗∗^ represents *p* < 0.001.

To comprehensively evaluate the immune condition in IC/BPS samples, we assessed the immunological characteristics of IC/BPS based on the infiltration of immune cells. As shown in Figure 5(a), both adaptive and innate infiltrating immune cells were significantly enriched in the IC/BPS cohort relative to the normal control group. In addition, correlation analysis showed impressive relationships between 28 immune cells that had penetrated the tissue (Figure 5(b)). Furthermore, the logistic regression model revealed that the majority of immune cells were positively associated with the diagnosis of IC (Figure 5(c)). Moreover, as anticipated, four of the five IC/BPS characteristic genes (RASGRP1, PROK2, PPBP, and CR2) demonstrated direct positive interactions with immune cell infiltration, whereas only RBP4 demonstrated a negative correlation (Figure 5(e)), consistent with the low expression of RBP4 in the IC/BPS group (Figure 4(h)). Finally, the radar plot visualized the scores of infiltrated immune cells in which interestingly, we found that central memory CD4 T cells, plasmacytoid dendritic cells, and monocytes performed the highest scores in both cohorts (Figure 5(d)). These results reveal that IC/BPS signature genes may regulate immunological characteristics throughout the development and progression of the IC/BPS process.

### 3.5. Development of Immune Subtypes Based on Immune-Related IC/BPS-DEGs

**Figure 6:**
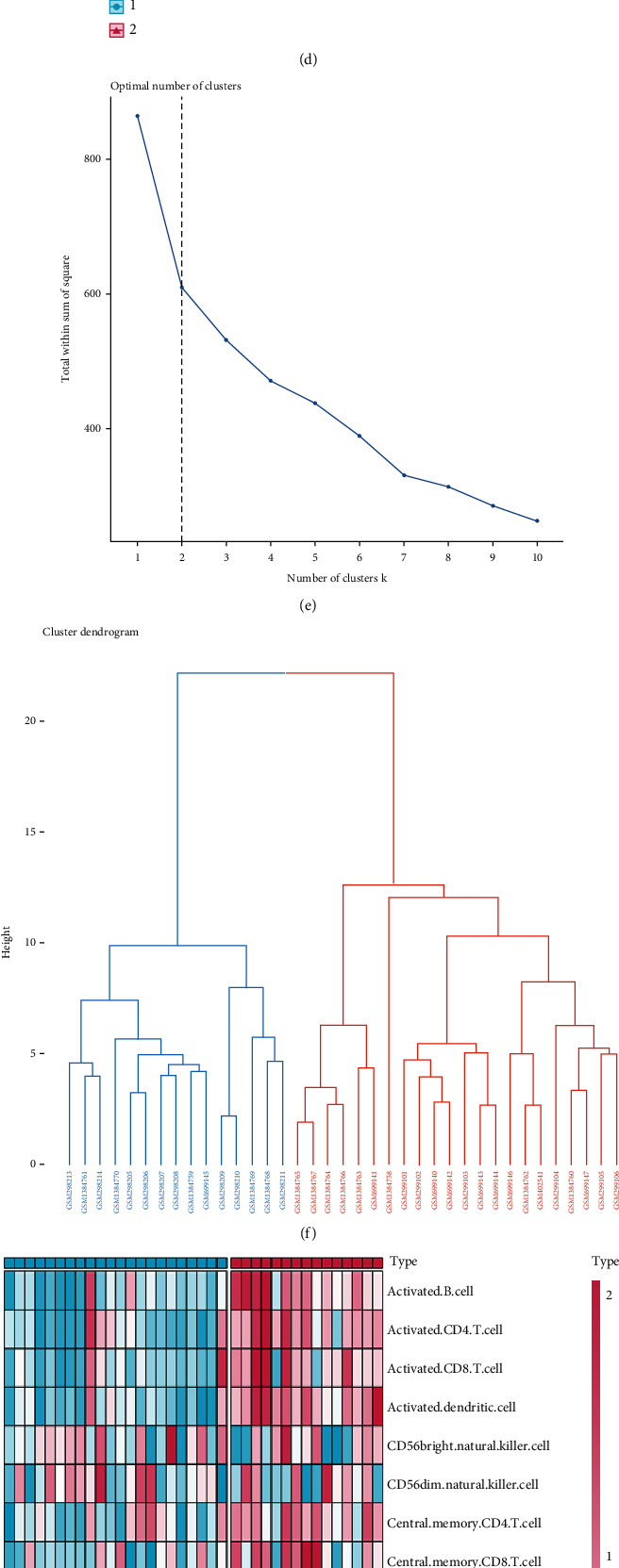
Development of immune subtypes based on immune-related IC-DEGs. (a) The consensus score matrix of IC samples when *k* = 2. (b) Consensus CDF curve when *k* = 2–7. (c) Relative alterations in the area under CDF curve. (d) PCA plots perform that all IC samples are categorized as two immune subtypes. (e, f) The optimal number of clusters determined by Nbclust. (g) Abundance of 28 infiltrated immune cells evaluated by ssGSEA for cluster1 and cluster2. (h) The heatmap performs the expression of immune-related IC-DEGs in IC immune clusters.

To further investigate the immunological characteristics of IC/BPS, we conducted the consensus cluster analysis on 37 IC/BPS cases based on the gene expression patterns of the immune-related IC/BPS-DEGs. We subsequently classified the IC/BPS samples into cluster1 and cluster2 immunological subtypes (Figures 6(a)–6(c)). PCA was utilized to illustrate the substantial distinctions between these two groups (Figure 6(d)). The ideal number (*k* = 2) of clusters was calculated by integrating the CDF curve, consensus score matrix, Nbclust, and PAC score (Figures 6(e) and 6(f)). As shown in Figure 6(h), immune-related IC/BPS-DEGs exhibited substantial subtype heterogeneity. In addition, the heatmap revealed the difference in infiltrating immune cells estimated by the ssGSEA method, with cluster2 displaying an abundance of immune cells (Figure 6(g)).

**Figure 7:**
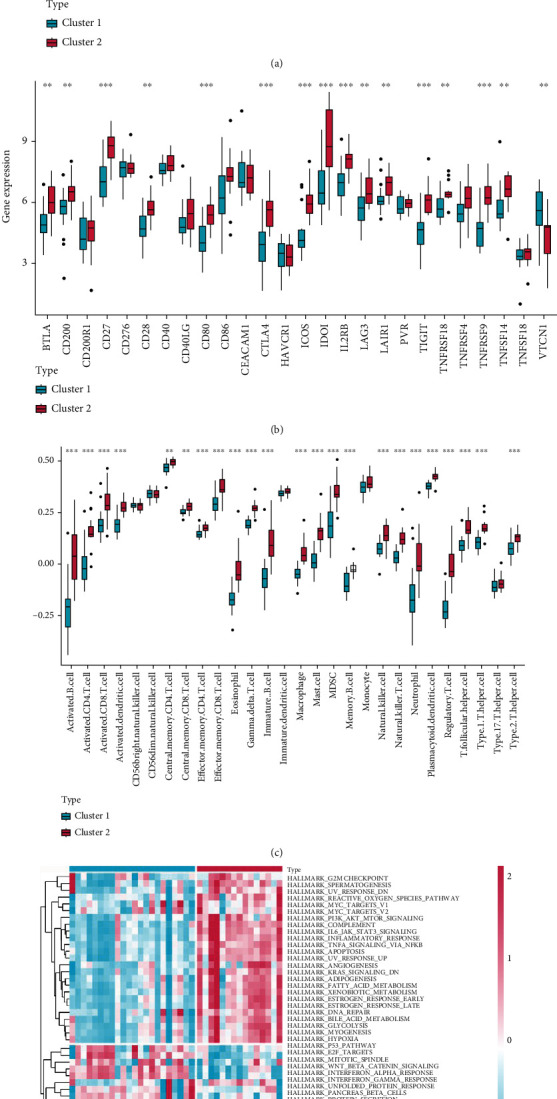
Immunological features and molecular mechanisms of IC immune subtypes. (a) Box plots perform the expression of IC characteristic genes in IC immune subtypes. (b) Box plots perform the expression of immune checkpoint-related genes in IC immune subtypes. (c) Box plots perform the abundance of 28 infiltrated immune cells evaluated by ssGSEA in IC immune subtypes. (d) The heatmap shows the enrichment levels of hallmark gene sets in IC immune subtypes.

We found that all of the gene characteristics of IC/BPS, except RBP4, were strongly expressed in the cluster2 subtype. This may be associated with the fact that there is a negative correlation between RBP4 and the number of immune cell infiltration (Figure 7(a)). In addition, we discovered that the majority of immune checkpoint inhibitor (ICI) genes were significantly elevated in the cluster2 subtype (Figure 7(b)), which is consistent with the greater amount of immune cell infiltration in the cluster2 subtype (Figure 7(c)). Based on the GSVA algorithm, the cluster2 subtype had significant immunological activation (G2M-checkpoint, MYC targets, PI3K-AKT-mTOR signaling pathway, and inflammatory response), Figure 5(d). In conclusion, IC/BPS samples were separated into two distinct immune subtypes. Cluster1 was found to have a low immune-infiltrating subtype, while cluster2 was found to have a high immune-infiltrating subtype.

### 3.6. Identification and Validation of Small-Molecule Drugs

**Figure 8:**
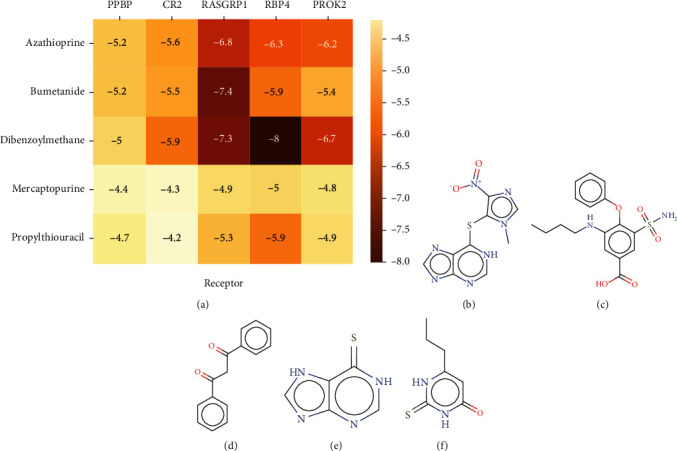
Identification and validation of small-molecule drugs. (a) The heatmap performs the lowest binding energy for molecular docking. (b–f) Chemical structure depiction of the potential small-molecule drugs. (b) Azathioprine, (c) bumetanide (d) dibenzoylmethane, (e) mercaptopurine, and (f) propylthiouracil.

**Figure 9:**
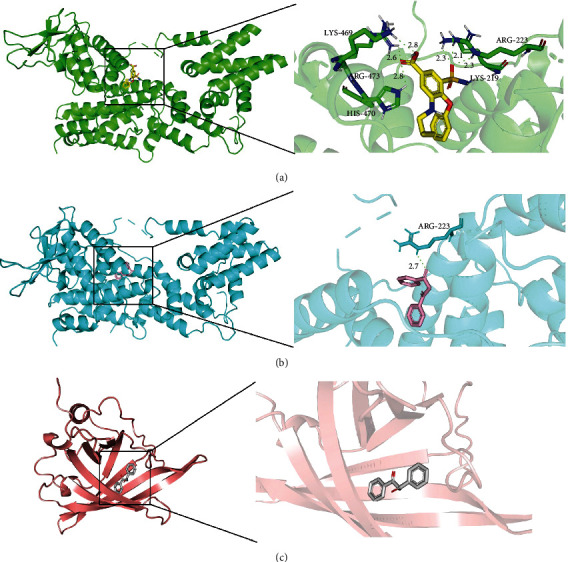
Docking diagram of potential small-molecule drugs with targets. (a) bumetanide-RASGRP1. (b) dibenzoylmethane-RASGRP1. (c) dibenzoylmethane-RBP4.

Using the CMap database, prospective small-molecule medicines for IC/BPS therapy were predicted based on immune-related IC/BPS characteristic genes.  Figure 8(b) depicts the exact chemical structures of these five molecules. Subsequently, we performed molecular docking between small-molecule drugs and five immune-related IC/BPS characteristic genes based on AutoDock Vina software. The binding free energy indicates the degree of conformational stability. Lower binding free energy indicates more conformational stability. When the binding free energy is less than zero, the ligand spontaneously attaches to the receptor [23].  Figure 8(a) reveals that the binding free energy of bumetanide and RASGRP1 is -7.4, showing that bumetanide's active component has a high affinity for RASGRP1. As shown in Figures 9(a)–9(c), the putative docking targets for small-molecule drugs were displayed. Results indicated that bumetanide performs its biological activity most likely by binding to RASGRP1 and establishing hydrogen bonds with five amino acid positions near the active site: LYS469, ARG473, HIS470, ARG223, and LYS219 (Figure 9(a)).

## 4. Discussion

Interstitial cystitis/bladder pain syndrome IC/BPS is a devastating illness that is characterized by severe pelvic pain and symptoms affecting the urinary system. Because of the complex nature of its disease process, IC/BPS does not have any reliable characteristic biomarkers or therapeutic methods [24–26]. Previous studies have shown that abnormal immunity is a significant histological feature in IC/BPS [27]; both innate and adaptive immune mechanisms may influence the pathogenesis and progression of IC/BPS [11, 12, 28]. Therefore, exploring the roles of infiltrated immune cells and immune-related genes during the progression of IC/BPS is important.

Our study combined the gene expression profiles of 37 IC/BPS samples and 20 normal control samples by integrating publicly available datasets (GSE11783, GSE11839, GSE28242, and GSE57560) obtained from the GEO database. The differential analysis then identified 55 upregulated DEGs and 62 downregulated DEGs in the IC/BPS cohort. The differential analysis found that the IC/BPS cohort had 62 DEGs that had decreased in expression and 55 DEGs that had increased in expression. Five immune-related IC/BPS characteristic genes were chosen for further study based on the PPI network and three different machine learning techniques (RASGRP1, PPBP, RBP4, CR2, and PROS2). In order to examine the characteristic value of these genes in IC/BPS, a nomogram and ROC curves were also constructed. The results showed that each of these genes could predict the development of IC/BPS accurately. In addition, GO and KEGG enrichment analysis uncovered evidence that these genes are associated with antimicrobial humoral response, cytokine-cytokine receptor interaction, and the IL-17 signaling pathway. These results align with those obtained in a prior investigation of IC/BPS, suggesting a direct connection between the distinctive genes and the immunological infiltration seen in IC/BPS [29–32].

It is believed that aberrant expression of RASGRP1 plays an important part in the development of autoimmunity. According to Baars et al. [33], dysregulation of RASGRP1 often takes place in activated T cells and may, in a dose-dependent way, affect TCR-induced signaling as well as thymocyte selection. The immune response hypothesis of IC/BPS etiology [27], which is partly explained by this finding, suggests that RASGRP1 may have potential significance to the immunological environment of IC/BPS. It is currently known that PPBP, which is an activator of neutrophils that is released by the megakaryocyte lineage, may be found expressing in a variety of cell types, which suggests that it may have a possible function in the establishment of IC/BPS immunological characteristics [34]. The expression of RBP4 is negatively linked with infiltrating macrophages, T cells, B cells, neutrophils, and dendritic cells [35], which is consistent with our results. Additional evidence demonstrates that RBP4 is an essential regulator of immune microenvironment homeostasis. Furthermore, CR2 is often present on B cells, follicular dendritic cells, and a fraction of T cells, which may affect B cell activity on many levels [36, 37], thereby modulating the immunological response to IC/BPS.

Peng et al. [6] have established that the immunological milieu of IC/BPS encompasses diverse innate and adaptive immune cells. This adds to the growing body of evidence suggesting that inflammation and immunity play a significant role in the evolution of IC/BPS. In our research, the ssGSEA algorithm was used to determine the number of immune cells that had been infiltrated, with the goal of doing an extensive evaluation of the immune cell infiltration that occurs in individuals who have IC/BPS. Innate and adaptive immune cell infiltration was significantly higher in the IC/BPS samples compared to the normal control samples. Moreover, the logistical model uncovered the characteristic value of infiltrating immune cells in predicting IC/BPS. Furthermore, the majority of the gene characteristics of IC/BPS have substantial positive correlations with the immune cells that have invaded the tissue, suggesting that these genes may influence immune activation as the condition of IC/BPS progresses.

As mentioned above, we found five potential small-molecular compounds that can effectively reverse the altered expression of the immune-related IC/BPS characteristic genes and improve IC/BPS through AutoDock Vina software. Among the five compounds, azathioprine is a synthetic purine and has a steroid-sparing effect [38]. Dibenzoylmethane is a beta-diketone analog of curcumin and is used in the treatment of diabetes-induced renal injury through its anti-inflammatory and antioxidant effects [39]. Mercaptopurine is an analog of the natural purines and has been widely used in the immunosuppressive therapy in interstitial lung disease [40]. As an effective inhibitor of thyroid iodide peroxidase, propylthiouracil can catalyze the biosynthesis of thyroid hormone from the initial step and have been extensively used for patients with hyperthyroidism [41]. Nevertheless, previous reports have not performed the effectiveness of these drugs in treating IC/BPS.

The previous study has shown that fibrosis, a typical pathological hallmark of many chronic inflammatory illnesses, plays a vital role in the course of IC/BPS, a disease classified as a chronic inflammatory disease [42]. As a result, developing effective therapeutics for bladder tissue fibrosis could be a feasible therapeutic target for IC/BPS [43]. Because bumetanide is an inhibitor of a member of the solute carrier family, it is a loop diuretic that is safe to use for the treatment of hypervolemia and has very minimal side effects. This finding may make its utilization in antifibrotic therapy more feasible [44–46]. It was demonstrated in an in vitro experiment by Zuo et al. [47] that bumetanide could inhibit collagen biosynthesis in fibroblasts by targeting the interaction of CRTH2 and LARP6, resulting in the treatment of organ fibrosis, suggesting that bumetanide may alleviate symptoms of IC/BPS patients by inhibiting bladder fibrosis. The molecular docking method was applied to bumetanide and the IC/BPS signature gene RASGRP1, where the low binding free energy performed good affinity between ligand and binding sites, suggesting that bumetanide may be a potent inhibitor.

To further investigate the immune cell infiltration of IC/BPS, we conducted a consensus cluster analysis of IC samples based on the immune-related IC/BPS-DEGs, dividing all IC/BPS samples into two immunological subtypes. We discovered that cluster2 (high immune-infiltrating subtype) exhibits a much greater quantity of immune cell infiltration and upregulation of ICI-related genes than cluster1 (low immune-infiltrating subtype). In addition, the functional enrichment analysis suggested that cluster2 has more immunological activation. Consequently, our findings suggest that the immunological subtype we suggested partly represents the immune landscape of IC/BPS, which may provide substantial insight into the early identification and successful treatment of these individuals. Despite the fact that our findings were based solely on machine learning algorithms and bioinformatics validation, we systematically explored the immune landscape of IC/BPS for the first time, identified and validated the characteristic value of immune-related IC/BPS signature genes, selected and verified potential small-molecule drugs, and proposed the IC/BPS immune subtypes. Future research will use more prospective studies to investigate the probable characteristic and therapeutic relevance of immune-related IC/BPS characteristic genes and possible small-molecule medicines.

## Data Availability

The original data presented in this study can be found in online repositories. Contact the corresponding authors for further inquiries.

## Supplementary Materials

Supplementary Materials Table S1: basic information of the datasets used in this article. Table S2: the gene list of the immune-related genes and immune checkpoint blockade-related genes.
