# Supplementary material for: Identification of Immune-Related Genes and Small-Molecule Drugs in Interstitial Cystitis/Bladder Pain Syndrome Based on the Integrative Machine Learning Algorithms and Molecular Docking
Source: J Immunol Res. 2022 Dec 28;2022:2069756. doi: 10.1155/2022/2069756 (PMC9812613; doi:10.1155/2022/2069756)
Supplement: Supplementary Materials — Table S1: basic information of the datasets used in this article. Table S2: the gene list of the immune-related genes and immune checkpoint blockade-related genes. [file 2069756.f1.zip › Table S1.pdf]

Basic information of GEO datasets

GSE series	Type	Sample size<br>Normal control	 Interstitial cystitis/bladder pain syndrome	Platform
GSE11783	mRNA	6	10	GPL570
GSE11839	mRNA	6	6	GPL570
GSE28242	mRNA	5	8	GPL6244
GSE57560	mRNA	3	13	GPL16699
