# Supplementary material for: Identification of Immune-Related Genes and Small-Molecule Drugs in Interstitial Cystitis/Bladder Pain Syndrome Based on the Integrative Machine Learning Algorithms and Molecular Docking
Source: J Immunol Res. 2022 Dec 28;2022:2069756. doi: 10.1155/2022/2069756 (PMC9812613; doi:10.1155/2022/2069756)
Supplement: Supplementary Materials — Table S1: basic information of the datasets used in this article. Table S2: the gene list of the immune-related genes and immune checkpoint blockade-related genes. [file 2069756.f1.zip › Table S2.pdf]

Immune-related obtained from Immport database

AZGP1
B2M
CALR
CANX
CD1A
CD1B
CD1C
CD1D
CD1E
CD4
CD8A
CD8B
CD74
CREB1
CTSB
CTSE
CTSL
CTSS
FCER1G
FCGRT
PDIA3
HFE
HLA-A
HLA-B
HLA-C
HLA-DMA
HLA-DMB
HLA-DOA
HLA-DOB
HLA-DPA1
HLA-DPB1
HLA-DQA1
HLA-DQA2
HLA-DQB1
HLA-DRA
HLA-DRB1
HLA-DRB3
HLA-DRB4
HLA-DRB5
HLA-E
HLA-F
HLA-G
HLA-H
MR1
HSPA1A
HSPA1B
HSPA1L
HSPA2
HSPA4
HSPA5
HSPA6
HSPA8
HSP90AA1
HSP90AB1
ICAM1

IFNA1
IFNA2
IFNA4
IFNA5
IFNA6
IFNA7
IFNA8
IFNA10
IFNA13
IFNA14
IFNA16
IFNA17
IFNA21
IFNG
KIR2DL1
KIR2DL2
KIR2DL3
KIR2DL4
KIR2DS1
KIR2DS3
KIR2DS4
KIR2DS5
KIR3DL1
KIR3DL2
KLRC1
KLRC2
KLRC3
KLRD1
LTA
CIITA
MICA
MICB
NFYA
NFYB
NFYC
LGMN
PSMB8
PSMC1
PSMC2
PSMC3
PSMC4
PSMC5
PSMC6
PSMD1
PSMD2
PSMD3
PSMD4
PSMD5
PSMD7
PSMD8
PSMD10
PSMD11
PSMD13
PSME1
PSME2
RELB
RFX5
RFXAP

SLC10A2
TAP1
TAP2
TAPBP
THBS1
SEM1
KLRC4
AP3B1
RFXANK
PSMD6
PSME3
PSMD14
CLEC4M
IFI30
PROCR
ADRM1
ECPAS
TRPC4AP
CD209
UBXN1
ERAP1
TAPBPL
KIR2DL5A
ERAP2
ULBP3
ULBP2
ULBP1
KIR3DL3
RAET1E
RAET1L
UBR1
RAET1G
PDIA2
HAMP
PI3
CAMP
DEFB4A
PPBP
REG3G
CXCL14
CXCL16
SLPI
CXCL8
CXCL10
CXCL9
CXCL5
CXCL11
CXCL6
CXCL1
CXCL12
CXCL13
CXCL2
PF4
XCL1
CXCL3
DEFB103B
CCL13
CCL1

DEFB1
CCL8
ELANE
DEFB103A
DEFA3
DEFA1
TMSB10
DEFA6
DEFA5
DEFA4
LCN2
LCN1
COLEC10
BPI
S100A9
S100A8
DCD
LCN6
S100A12
HTN3
LCN8
DEFA1B
CCR10
CELA1
DEFB106A
PENK
BPIFC
MMP12
BPIFB6
LEAP2
SFTPD
LCN9
BPIFB2
PTGDS
TMSB4X
PGLYRP1
ZC3HAV1
TMSB15A
S100B
S100A13
S100A6
DEFB119
DEFB107A
DEFB105A
SERPIND1
DEFB129
DEFB127
S100P
S100A7
DEFB104A
DEFB126
DEFB106B
DEFB104B
DEFB107B
PGLYRP3
PGLYRP2
S100A10
S100A2

DEFB125
DEFB123
DEFB105B
DEFB132
BPIFB3
LCN12
PGLYRP4
S100A11
S100A5
S100A3
S100A1
DEFB128
DEFB108B
HTN1
LMBR1L
S100A7A
DEFB118
COLEC12
TMSB4Y
DEFB131A
DEFB134
DEFB130A
DEFB124
DEFB121
DEFB116
DEFB115
DEFB114
DEFB113
DEFB112
DEFB110
TMSB15B
DEFB133
S100Z
MAVS
TMSB4XP8
S100A14
LCN10
S100A16
DEFB136
DEFB135
DEFB117
ZC3HAV1L
S100A7L2
MBL3P
DEFB4B
BPIFB4
IFNAR1
AZU1
DEFB131B
DEFA1A3
LCN1P1
S100G
DEFA7P
DEFB130B
DEFB108F
DEFB131C
TCHHL1
TINAGL1

IFNGR1
SLC22A17
WFIKKN1
WFDC2
IL6
UMODL1
TGFB1
PF4V1
MMP9
ANOS1
TLR4
SPAG11B
A2M
NFKB1
APOBEC3G
FABP6
NOD2
MBL2
SFTPA1
RBP1
TLR2
SLC40A1
PLAU
IL1B
PAEP
HJV
MUC5AC
OBP2A
PLTP
MX1
DDX58
IFNL1
IRF3
SFTPA2
LPA
LBP
RBP4
NOX4
LTF
IFNB1
RBP5
FABP7
FABP5
FABP3
FABP2
FABP4
R3HDML
BPIFA3
BPIFB1
OASL
CRABP2
CRABP1
RBP7
DUOX1
OBP2B
RBP2
LCN15
CETP

FABP12
FABP9
BPIFA1
LCNL1
C8G
SPAG11A
PI15
NOX1
PMP2
APOD
ORM2
ORM1
TNF
CTSG
PRTN3
MAPK1
PML
AEN
CYBB
BPIFA2
ISG20
BCL3
ISG20L2
NOX5
NOX3
DUOX2
TLR3
TFRC
IFIH1
LRP1
TRIM5
IDO1
GDF15
NEDD4
ADIPOQ
STAT3
STAT1
IFNL2
SOCS3
SEMG1
TNFSF10
CCL20
SOCS1
RNASEL
IRF1
IL15
APOBEC3F
PLAAT4
CHIT1
CD40
TLR7
PPIA
ZYX
NLRX1
PGC
VEGFA
IKBKE
ISG15

DHX58
TNFAIP3
TFR2
FCN2
MUC4
F2R
ELN
IL27
MAPT
LYZ
CCL5
LEP
CYLD
KLKB1
CST4
CSRP1
MAPK14
JUN
ITGAV
IRF5
CCR6
IL12B
TLR8
GNLY
CD81
EIF2AK2
APOM
CACYBP
NOD1
MAPK8
MAPK3
BST2
BPHL
PLA2G2A
GRN
NEWENTRY
PDGFRA
GNAI1
WNT5A
FURIN
ADAR
TYK2
NOS2
TRAF3
TPT1
TPM2
NEO1
AHNAK
TLR1
TK2
PRDX2
MX2
FGF2
FGA
TCF7L2
F2RL1
TKFC
MSR1

NFKBIZ
LMBR1
EPPIN
SRC
MPO
ELAVL1
ROBO3
SP1
SOD1
PDF
DLL4
ECD
SLC11A1
DMBT1
STING1
SKIV2L
SEMG2
DES
DCK
DAXX
TNFRSF10A
TNFRSF10B
EED
CCL4
LIMS1
LALBA
APOBEC3H
TMPRSS6
SPINK5
MARCO
BECN1
TNFSF11
KNG1
CSK
KLRK1
KCNH2
JUND
JAK1
CLDN4
CCL28
RNASE3
RN7SL1
IRF7
IREB2
ILK
IL18
IL17A
LTB4R
APOBEC3A
MASP2
TRIM27
RELA
IL7R
IL1A
PTX3
IFNAR2
IFN1@
SYTL1

APOBEC3C
DDX17
PTGS2
HTR1A
SEPTIN7
CD40LG
CD14
MASP1
PROC
MAP2K2
MAP2K1
HRG
NDRG1
IRF9
TRIM22
LANCL1
PPP4C
HMOX1
HMGB1
RNASE7
ABCC4
HGF
HDAC1
IFNLR1
PLSCR1
BACH2
TANK
PIK3CG
ARRB1
RSAD2
STAB2
TBK1
PDYN
PDGFRB
PDCD1
PCSK2
PCSK1
ARG2
AQP9
FASLG
APOH
BIRC5
ANXA6
IL22
VTN
VIM
VCAM1
PRDX1
GFAP
GBP2
ALB
SLC29A3
OAS1
AGER
UNC93B1
TNFSF4
NOS1
ACTG1

ACTA1
ACO1
SERPINA3
CXCR1
CCL15
CCL14
CCL16
CCL19
CCL18
CCL17
CCL26
CCL22
CCR3
CCL4L1
ACKR2
CCR7
CCL27
CCR8
ACKR4
CCL2
CCL21
CCL7
CCL3
CCL11
CCR5
CCL23
CCL25
CCL3L3
CCL4L2
CCL3L1
CCR1
CCL24
XCL2
CXCR4
CXCR6
CCR4
TAFA5
TAFA3
TAFA4
TAFA1
TAFA2
CCL15-CCL14
PTK2B
IL4
CDH1
LTBP1
IL13
IL10
IL2
PPARG
FGR
MIF
CRP
JAK2
PTK2
PTGDR
CD86
HCK

VDR
OLR1
GRK2
TXK
RNASE2
CD79A
CD79B
LYN
SYK
BTK
BLNK
VAV3
VAV1
VAV2
RAC1
RAC2
RAC3
PPP3CA
PPP3CB
PPP3CC
CHP1
PPP3R1
PPP3R2
CHP2
NFAT5
NFATC1
NFATC2
NFATC3
NFATC4
HRAS
KRAS
NRAS
FOS
CARD11
BCL10
MALT1
CHUK
IKBKB
IKBKG
NFKBIA
NFKBIB
NFKBIE
CD19
CR2
PIK3R5
PIK3R1
PIK3R2
PIK3R3
PIK3CA
PIK3CB
PIK3CD
AKT3
AKT1
AKT2
GSK3B
INPP5D
CD22
CD72

PTPN6
LILRB3
FCGR2B
RASGRP3
PLCG2
PRKCB
IFITM1
IGH
IGHA1
IGHA2
IGHD
IGHD1-1
IGHD1-14
IGHD1-20
IGHD1-26
IGHD1-7
IGHD2-15
IGHD2-2
IGHD2-21
IGHD2-8
IGHD3-10
IGHD3-16
IGHD3-22
IGHD3-3
IGHD3-9
IGHD4-11
IGHD4-17
IGHD4-23
IGHD4-4
IGHD5-12
IGHD5-18
IGHD5-24
IGHD5-5
IGHD6-13
IGHD6-19
IGHD6-25
IGHD6-6
IGHD7-27
IGHE
IGHG1
IGHG2
IGHG3
IGHG4
IGHJ1
IGHJ2
IGHJ3
IGHJ4
IGHJ5
IGHJ6
IGHM
IGHV1-18
IGHV1-2
IGHV1-24
IGHV1-3
IGHV1-45
IGHV1-46
IGHV1-58
IGHV1-69

IGHV1-8
IGHV1-38-4
IGHV1-69-2
IGHV2-26
IGHV2-5
IGHV2-70
IGHV3-11
IGHV3-13
IGHV3-15
IGHV3-16
IGHV3-20
IGHV3-21
IGHV3-23
IGHV3-30
IGHV3-30-3
IGHV3-30-5
IGHV3-33
IGHV3-35
IGHV3-38
IGHV3-43
IGHV3-48
IGHV3-49
IGHV3-53
IGHV3-64
IGHV3-66
IGHV3-7
IGHV3-72
IGHV3-73
IGHV3-74
IGHV3-9
IGHV3-38-3
IGHV3-69-1
IGHV4-28
IGHV4-30-1
IGHV4-30-2
IGHV4-30-4
IGHV4-31
IGHV4-34
IGHV4-39
IGHV4-4
IGHV4-59
IGHV4-61
IGHV4-38-2
IGHV5-51
IGHV5-10-1
IGHV6-1
IGHV7-4-1
IGHV7-81
IGK
IGKC
IGKDEL
IGKJ
IGKJ1
IGKJ2
IGKJ3
IGKJ4
IGKJ5
IGKV@

IGKV1-12
IGKV1-13
IGKV1-16
IGKV1-17
IGKV1-27
IGKV1-33
IGKV1-37
IGKV1-39
IGKV1-5
IGKV1-6
IGKV1-8
IGKV1-9
IGKV1D-12
IGKV1D-13
IGKV1D-16
IGKV1D-17
IGKV1D-33
IGKV1D-37
IGKV1D-39
IGKV1D-42
IGKV1D-43
IGKV1D-8
IGKV2-24
IGKV2-28
IGKV2-30
IGKV2-40
IGKV2D-24
IGKV2D-28
IGKV2D-29
IGKV2D-30
IGKV2D-40
IGKV3-11
IGKV3-15
IGKV3-20
IGKV3-7
IGKV3D-11
IGKV3D-15
IGKV3D-20
IGKV3D-7
IGKV4-1
IGKV5-2
IGKV6-21
IGKV6D-21
IGKV6D-41
IGL
IGLC1
IGLC2
IGLC3
IGLC6
IGLC7
IGLJ
IGLJ1
IGLJ2
IGLJ3
IGLJ4
IGLJ5
IGLJ6
IGLJ7

IGLV@
IGLV1-36
IGLV1-40
IGLV1-44
IGLV1-47
IGLV1-50
IGLV1-51
IGLV10-54
IGLV11-55
IGLV2-11
IGLV2-14
IGLV2-18
IGLV2-23
IGLV2-33
IGLV2-8
IGLV3-1
IGLV3-10
IGLV3-12
IGLV3-16
IGLV3-19
IGLV3-21
IGLV3-22
IGLV3-25
IGLV3-27
IGLV3-32
IGLV3-9
IGLV4-3
IGLV4-60
IGLV4-69
IGLV5-37
IGLV5-39
IGLV5-45
IGLV5-48
IGLV5-52
IGLV6-57
IGLV7-43
IGLV7-46
IGLV8-61
IGLV9-49
C3
C5
CCL3P1
CKLF
CMA1
CX3CL1
CXCL17
CCN1
EDN1
EDN2
EDN3
FGF10
LECT2
PPBPP1
PROK2
SAA1
SAA2
SBDS
SEMA3A

SEMA3B
SEMA3C
SEMA3D
SEMA3E
SEMA3F
SEMA3G
SEMA4A
SEMA4B
SEMA4C
SEMA4D
SEMA4F
SEMA4G
SEMA5A
SEMA5B
SEMA6A
SEMA6B
SEMA6C
SEMA6D
SEMA7A
SLIT1
SLIT2
TNC
TYMP
C5AR1
CCR9
CCRL2
CMKLR1
CX3CR1
CXCR3
CXCR5
ACKR3
CYSLTR1
CYSLTR2
ACKR1
EDNRA
EDNRB
FPR1
FPR2
GPR17
GPR32
GPR33
PTGDR2
C5AR2
CXCR2
LTB4R2
PLAUR
PLXNA1
PLXNA2
PLXNA3
PLXNA4
PLXNB1
PLXNB2
PLXNB3
PLXNC1
PLXND1
PTAFR
ROBO1
ROBO2

RXFP3
XCR1
ADM
ADM2
AGRP
AGT
AMBN
AMELX
AMH
ANGPTL5
ANGPTL7
APLN
AREG
MANF
CDNF
ARTN
AVP
BDNF
BMP1
BMP10
BMP15
BMP2
BMP3
BMP4
BMP5
BMP6
BMP7
BMP8A
BMP8B
BTC
MYDGF
CALCA
CALCB
CAT
CCK
CD320
CD70
ADA2
CER1
CGA
CGB3
CGB1
CGB2
CGB5
CGB7
CGB8
CHGA
CHGB
CLCF1
CLEC11A
CMTM1
CMTM2
CMTM3
CMTM4
CMTM5
CMTM6
CMTM7
CMTM8

CNTF
CORT
CRH
CSF1
CSF2
CSF3
CSH1
CSH2
CSHL1
CSPG5
CTF1
CCN2
DKK1
EBI3
EGF
EPGN
EPO
EREG
ESM1
FAM3B
FAM3C
FAM3D
FGF1
FGF11
FGF12
FGF13
FGF14
FGF16
FGF17
FGF18
FGF19
FGF20
FGF21
FGF22
FGF23
FGF3
FGF4
FGF5
FGF6
FGF7
FGF8
FGF9
VEGFD
FIGNL2
FLT3LG
FSHB
GAL
GALP
GAST
GCG
GDF1
GDF10
GDF11
GDF2
GDF3
GDF5
GDF6
GDF7

GDF9
GDNF
GH1
GH2
GHRH
GHRL
GIP
GKN1
GMFB
GMFG
GNRH1
GNRH2
GPHA2
GPHB5
GPI
GREM1
GREM2
GRP
GUCA2A
HBEGF
HDGF
HDGFL3
IAPP
IFNE
IFNK
IFNW1
IGF1
IGF2
IL11
IL12A
IL16
IL17B
IL17C
IL17D
IL17F
IL19
IL1F10
IL36RN
IL36A
IL37
IL36B
IL36G
IL1RN
IL20
IL21
IL23A
IL24
IL25
IL26
IFNL3
IL3
IL31
IL32
IL33
IL34
IL5
IL6ST
IL7

IL9
INHA
INHBA
INHBB
INHBC
INHBE
INS
INS-IGF2
INSL3
INSL4
INSL5
INSL6
JAG1
JAG2
FGF7P6
FGF7P3
KITLG
KL
LACRT
LEFTY1
LEFTY2
LHB
LIF
LRSAM1
LTB
LTBP2
LTBP3
LTBP4
MDK
MIA
MLN
MSTN
NAMPT
NDP
NENF
NGF
NMB
NODAL
CCN3
NPFF
NPPA
NPPB
NPPC
NPY
NRG1
NRG2
NRG3
NRG4
NRTN
NTF3
NTF4
NTS
NUDT6
OGN
OSGIN1
OSM
OSTN
OXT

ENDOU
PDGFA
PDGFB
PDGFC
PDGFD
PDGFRL
PGF
PMCH
PNOC
POMC
PPBPP2
PPY
PRL
PRLH
PROK1
PSPN
PTH
PTH2
PTHLH
PTN
PYY
QRFP
RABEP1
RABEP2
REG1A
RETN
RETNLB
RLN1
RLN2
RLN3
SCG2
SCGB3A1
SCT
AIMP1
SECTM1
SLURP1
SPP1
SST
STC1
STC2
TAC1
TDGF1
TDGF1P3
TG
TGFA
TGFB2
TGFB3
THPO
TNFRSF11B
TNFSF12
TNFSF13
TNFSF13B
TNFSF14
TNFSF15
TNFSF18
TNFSF8
TNFSF9
TOR2A

TRH
TSHB
TSLP
TXLNA
UCN
UCN2
UCN3
UTS2
UTS2B
VEGFB
VEGFC
VGF
VIP
ACVR1B
ACVR1C
ACVR2A
ACVR2B
ACVRL1
ADCYAP1R1
ADIPOR1
ADIPOR2
ADRB1
ADRB2
AGTR1
AGTR2
AMHR2
ANGPT1
ANGPT4
ANGPTL1
ANGPTL2
ANGPTL3
ANGPTL4
ANGPTL6
APLNR
AR
AVPR1A
AVPR1B
AVPR2
BMPR1A
BMPR1B
BMPR2
BRD8
C3AR1
CALCR
CALCRL
CNTFR
CRHR1
CRHR2
CRIM1
CRLF1
CRLF2
CRLF3
CSF1R
CSF2RA
CSF2RB
CSF3R
EGFR
ENG

EPOR
ESR1
ESR2
ESRRA
ESRRB
ESRRG
FGFR1
FGFR2
FGFR3
FGFR4
FGFRL1
FLT1
FLT3
FLT4
FSHR
GALR2
GALR3
GCGR
GHR
GHRHR
GHSR
GIPR
GLP1R
GLP2R
GNRHR
GPER1
HNF4A
HNF4G
HTR3A
HTR3B
HTR3C
HTR3D
HTR3E
IFNGR2
IGF1R
IGF2R
IL10RA
IL10RB
IL11RA
IL12RB1
IL12RB2
IL13RA1
IL13RA2
IL15RA
IL2RB
IL17RA
IL17RB
IL17RC
IL17RD
IL17RE
IL18R1
IL18RAP
IL1R1
IL1R2
IL1RAP
IL1RL1
IL1RL2
IL20RA

IL20RB
IL21R
IL22RA1
IL22RA2
IL23R
IL27RA
IL2RA
IL2RG
IL31RA
IL3RA
IL4R
IL5RA
IL6R
IL9R
INSR
KDR
LEPR
LGR4
LGR5
LGR6
LHCGR
LIFR
LTBR
MC1R
MC2R
MC3R
MC4R
MCHR1
MCHR2
MET
MLNR
MPL
MTNR1A
MTNR1B
NGFR
NMBR
NPR1
NPR3
NR0B1
NR0B2
NR1D1
NR1D2
NR1H2
NR1H3
NR1H4
NR1I2
NR1I3
NR2C1
NR2C2
NR2E1
NR2E3
NR2F1
NR2F2
NR2F6
NR3C1
NR3C2
NR4A1
NR4A2

NR4A3
NR5A1
NR5A2
NR6A1
NRP1
NRP2
OGFR
OPRD1
OPRK1
OPRL1
OPRM1
OSMR
OXTR
PGR
PGRMC2
PPARA
PPARD
PRLHR
PRLR
PTGER1
PTGER2
PTGER3
PTGER4
PTGFR
PTH1R
PTH2R
RARA
RARB
RARG
RORA
RORB
RORC
RXFP1
RXFP2
RXRA
RXRB
RXRG
S1PR1
S1PR2
SCTR
SDC1
SDC2
SDC3
SDC4
SORT1
SSTR1
SSTR2
SSTR5
ST2
TACR1
TEK
TGFBR1
TGFBR2
TGFBR3
THRA
THRB
TIE1
TNFRSF10C

TNFRSF10D
TNFRSF11A
TNFRSF12A
TNFRSF13B
TNFRSF13C
TNFRSF14
TNFRSF17
TNFRSF18
TNFRSF19
TNFRSF1A
TNFRSF1B
TNFRSF21
TNFRSF25
TNFRSF4
TNFRSF6B
TNFRSF8
TNFRSF9
TRHR
TSHR
TUBB3
VIPR1
VIPR2
PTPN11
ICAM2
ITGAL
ITGB2
PAK1
NCR2
TYROBP
LCK
FCGR3A
FCGR3B
NCR1
NCR3
CD247
ZAP70
LCP2
LAT
PLCG1
SH3BP2
FYN
SHC2
SHC4
SHC3
SHC1
GRB2
SOS1
SOS2
ARAF
BRAF
RAF1
HCST
CD48
CD244
PRKCA
PRKCG
SH2D1B
SH2D1A

FAS
GZMB
PRF1
CASP3
BID
CD3D
CD3E
CD3G
PTPRC
ITK
TEC
NCK1
NCK2
GRAP2
PAK2
PAK3
PAK4
PAK6
PAK5
RHOA
CDC42
CD28
ICOS
MAP3K8
MAP3K14
CTLA4
CBLC
CBL
CBLB
CDK4
RASGRP1
PDK1
PRKCQ
TRAC
TRAJ1
TRAJ2
TRAJ3
TRAJ4
TRAJ5
TRAJ6
TRAJ7
TRAJ8
TRAJ9
TRAJ10
TRAJ11
TRAJ12
TRAJ13
TRAJ14
TRAJ15
TRAJ16
TRAJ17
TRAJ18
TRAJ19
TRAJ20
TRAJ21
TRAJ22
TRAJ23
TRAJ24

TRAJ25
TRAJ26
TRAJ27
TRAJ28
TRAJ29
TRAJ30
TRAJ31
TRAJ32
TRAJ33
TRAJ34
TRAJ35
TRAJ36
TRAJ37
TRAJ38
TRAJ39
TRAJ40
TRAJ41
TRAJ42
TRAJ43
TRAJ44
TRAJ45
TRAJ46
TRAJ47
TRAJ48
TRAJ49
TRAJ50
TRAJ52
TRAJ53
TRAJ54
TRAJ56
TRAJ57
TRAJ58
TRAJ59
TRAJ61
TRAV1-1
TRAV1-2
TRAV2
TRAV3
TRAV4
TRAV5
TRAV7
TRAV8-1
TRAV8-2
TRAV8-3
TRAV8-4
TRAV8-6
TRAV8-7
TRAV9-1
TRAV9-2
TRAV10
TRAV12-1
TRAV12-2
TRAV12-3
TRAV13-1
TRAV13-2
TRAV14DV4
TRAV16
TRAV17

TRAV18
TRAV19
TRAV20
TRAV21
TRAV22
TRAV23DV6
TRAV24
TRAV25
TRAV26-1
TRAV26-2
TRAV27
TRAV29DV5
TRAV30
TRAV34
TRAV35
TRAV36DV7
TRAV38-1
TRAV38-2DV8
TRAV39
TRAV40
TRAV41
TRBC1
TRBC2
TRBD1
TRBD2
TRBJ1-1
TRBJ1-2
TRBJ1-3
TRBJ1-4
TRBJ1-5
TRBJ1-6
TRBJ2-1
TRBJ2-2
TRBJ2-3
TRBJ2-4
TRBJ2-5
TRBJ2-6
TRBJ2-7
TRBV2
TRBV3-1
TRBV4-1
TRBV4-2
TRBV4-3
TRBV5-1
TRBV5-4
TRBV5-5
TRBV5-6
TRBV5-7
TRBV5-8
TRBV6-1
TRBV6-2
TRBV6-3
TRBV6-4
TRBV6-5
TRBV6-6
TRBV6-7
TRBV6-8
TRBV6-9

TRBV7-2
TRBV7-3
TRBV7-4
TRBV7-6
TRBV7-7
TRBV7-8
TRBV7-9
TRBV9
TRBV10-1
TRBV10-2
TRBV10-3
TRBV11-1
TRBV11-2
TRBV11-3
TRBV12-3
TRBV12-4
TRBV12-5
TRBV13
TRBV14
TRBV15
TRBV16
TRBV17
TRBV18
TRBV19
TRBV20-1
TRBV24-1
TRBV25-1
TRBV27
TRBV28
TRBV29-1
TRBV30
TRDC
TRDD1
TRDD2
TRDD3
TRDJ1
TRDJ2
TRDJ3
TRDJ4
TRDV1
TRDV2
TRDV3
TRGV9
TRGV8
TRGV5
TRGV4
TRGV3
TRGV2
TRGJP2
TRGJP1
TRGJP
TRGJ2
TRGJ1
TRGC2
TRGC1
TRAV6
